# Comparative transcriptome analysis reveals the response mechanism of Cf*-16*-mediated resistance to *Cladosporium fulvum* infection in tomato

**DOI:** 10.1186/s12870-020-2245-5

**Published:** 2020-01-20

**Authors:** Dongye Zhang, Yufang Bao, Yaoguang Sun, Huanhuan Yang, Tingting Zhao, Huijia Li, Chong Du, Jingbin Jiang, Jingfu Li, Libo Xie, Xiangyang Xu

**Affiliations:** 1 Laboratory of Genetic Breeding in Tomato, Key Laboratory of Biology and Genetic Improvement of Horticultural Crops (Northeast Region), Ministry of Agriculture and Rural Affairs, College of Horticulture and Landscape Architecture, Northeast Agricultural University, Harbin, 150030 China; 2 Horticultural Sub-Academy, Heilongjiang Academy of Agricultural Sciences, Harbin, 150069 China

**Keywords:** Comparative transcriptome, *Cladosporium fulvum*, Cf*-16* tomato, Molecular mechanism

## Abstract

**Background:**

Leaf mold disease caused by *Cladosporium fulvum* is a serious threat affecting the global production of tomato. Cf genes are associated with leaf mold resistance, including Cf*-16*, which confers effective resistance to leaf mold in tomato. However, the molecular mechanism of the Cf*-16*-mediated resistance response is largely unknown.

**Results:**

We performed a comparative transcriptome analysis of *C. fulvum*-resistant (cv. Ontario7816) and *C. fulvum*-susceptible (cv. Moneymaker) tomato cultivars to identify differentially expressed genes (DEGs) at 4 and 8 days post inoculation (dpi) with *C. fulvum*. In total, 1588 and 939 more DEGs were found in Cf*-16* tomato than in Moneymaker at 4 and 8 dpi, respectively. Additionally, 1350 DEGs were shared between the 4- and 8-dpi Cf*-16* groups, suggesting the existence of common core DEGs in response to *C. fulvum* infection. The up-regulated DEGs in Cf*-16* tomato were primarily associated with defense processes and phytohormone signaling, including salicylic acid (SA) and jasmonic acid (JA). Moreover, SA and JA levels were significantly increased in Cf*-16* tomato at the early stages of *C. fulvum* infection. Contrary to the previous study, the number of up-regulated genes in Cf*-16* compared to Cf*-10* and Cf*-12* tomatoes was significantly higher at the early stages of *C. fulvum* infection.

**Conclusion:**

Our results provide new insight into the Cf-mediated mechanism of resistance to *C. fulvum*, especially the unique characteristics of Cf*-16* tomato in response to this fungus.

## Background

Tomato (*Solanum lycopersicum* L.) is the second most important horticultural crop worldwide [1, 2] and an important model plant for fleshy fruit development and plant-pathogen interactions. Leaf mold disease caused by *Cladosporium fulvum* is considered to be one of the most devastating diseases in tomato*. C. fulvum* is a nonobligate, abiotrophic pathogenic fungus that infects foliage and occasionally petioles and stems [3–5]. Leaf mold has long been prevalent in many countries and caused serious economic loss, especially under high-temperature and high-humidity conditions [6]. Currently, the most effective way is to cultivate *C. fulvum*-resistant tomato varieties with resistance genes.

From a coevolutionary perspective, plants recognize and respond to pathogens in several phases. In the first phase, pathogen-associated molecular patterns (PAMPs) are recognized by pattern recognition receptors (PRRs) in plants, inducing PAMP-triggered immunity (PTI) and preventing pathogen colonization [7, 8]. In the second phase, successful pathogens bypass PTI and secrete effectors into plant cells, and the effector-triggered susceptibility response (ETS) ensues. In the third phase, plants gradually evolve to produce NB-LRR (nucleotide-binding site and leucine-rich repeat) proteins that directly or indirectly recognize specific pathogen effectors, and induce effector-triggered immunity (ETI). Finally, pathogens successfully infect plants and induce ETS by inhibiting or altering effectors that may be recognized by the plant and generating new effectors that cannot be recognized by the plant NB-LRRs. Simultaneously, plants again induce ETI by generating new R genes that encode proteins capable of identifying new effectors [9–11].

With respect to plant-pathogen interactions, different pathogens carry avirulence (*AVR*) genes corresponding to plant R genes and encode proteins that are recognized by effector proteins [12]. These proteins are secreted into the apoplastic space during infection and induce either compatible or incompatible interactions between fungi and infected plants [13]. Incompatible interactions (chlorosis) lead to the hypersensitive response (HR) when plants resist pathogens; compatible interactions occur when the pathogens can grow and ramify, causing necrosis in infected cells [14–17]. The tomato-*C. fulvum* interaction follows a typical gene-for-gene relationship, and the products of *C. fulvum*-resistance genes (Cf genes) in tomato specifically recognize the products encoded by the *AVR* genes in *C. fulvum*, leading to HR [18, 19]. At least 24 Cf genes have been reported since the discovery of the Cf*-1* gene in the 1930s [20, 21], and these genes have been introduced into cultivated tomatoes [22–30].

Transcriptome sequencing (RNA-Seq) has strongly accelerated research on host-pathogen interactions in plants such as rice [31], maize [32], cucumber [33], watermelon [34] and strawberry [35]. *Avr4/*Cf*-4-* and *Avr9/*Cf*-9-*dependent defense gene expression has been confirmed by cDNA-AFLP (cDNA-amplified fragment length polymorphism) analysis [36]. The *Avr5* gene has been cloned through a combined bioinformatic and RNA-Seq-based transcriptome sequencing approach [18]. Cf*-19-*, Cf*-12-* and Cf*-10-*mediated resistance to *C. fulvum* in tomato has been characterized using cDNA-AFLP and RNA-Seq analyses [37–39]. However, few transcriptomic studies have examined Cf*-16*-mediated resistance.

In this study, in addition to performing a transcriptomic analysis, we measured the endogenous hormone levels of resistant and susceptible tomato cultivars in response to *C. fulvum* infection. Our results are not only useful for understanding the mechanism of Cf-mediated resistance to *C. fulvum* infection but also providing a basis for cloning of the Cf*-16* gene.

## Results

### Microscopic analysis of *C. fulvum* invasion in two tomato cultivars

**Fig. 1:**
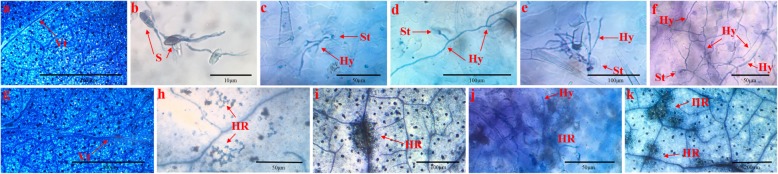
Trypan blue staining of tomato leaf tissues inoculated with *C. fulvum*. **a**-**f** Moneymaker leaf stained with lactophenol trypan blue at 0, 2, 4, 8 and 10–21 dpi, respectively. **g-k** Cf*-16* tomato leaf stained with lactophenol trypan blue at 0, 8, 10 and 12–21 dpi, respectively. Vt: vascular tissue, S: spore, St: stomata, Hy: hypha, HR: hypersensitive response

The *C. fulvum* infection process in Cf*-16* tomato or Moneymaker leaves was observed by light microscopy (Fig. 1). As shown in Fig. 1a and g, no difference was found between Cf*-16* tomato and Moneymaker at 0 dpi. Our results showed that conidiospores germinated at 2–3 dpi (Fig. 1b), with hyphae growing into the stomata in both Moneymaker and Cf*-16* tomato leaves at 4 dpi (Fig. 1c). The hyphae then emerged through the stomata of Moneymaker leaves at 8 dpi (Fig. 1d), with the growth and number of emergent hyphae continuing to increase through 10 dpi, and the last few infected cells starting to undergo necrosis at 10–21 dpi (Fig. 1e, f). In contrast, a small number of HR areas appeared at 8 dpi in Cf*-16* tomato (Fig. 1h), which had gradually grown at 10 dpi (Fig. 1i). In addition, hyphal growth was restricted to necrotic areas (Fig. 1j) until more necrotic lesions appeared in both mesophyll cells and leaf veins between 12 and 21 dpi (Fig. 1k). Obviously, plants carrying the Cf*-16* resistance gene showed a strong HR after infection with *C. fulvum*, whereas the susceptible plants (i.e., Moneymaker) showed continuous hyphal growth. Based on these observations, we collected samples from each treatment at 4 and 8 dpi for RNA-Seq and qRT-PCR (quantitative real-time PCR) analyses.

### Analysis of hormone response to *C. fulvum* infection

**Fig. 2:**
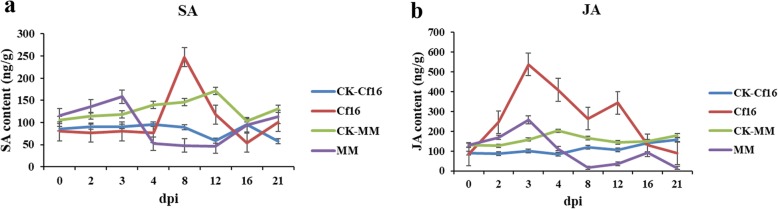
Fluctuations in SA and JA on different days post inoculation with *C. fulvum* in Cf*-16* tomato and Moneymaker. CK-Cf16: control group of Cf*-16* tomato, Cf16: inoculation group of *Cf-16* tomato, CK-MM: control group of Moneymaker, MM: inoculation group of Moneymaker

To explore hormone response to *C. fulvum* infection, HPLC-MS/MS (high-performance liquid chromatography-tandem mass spectrometry) was used to measure SA and JA levels. As shown in Fig. 2a, the SA content increased rapidly in Cf*-16* tomato after inoculation, peaking from 4 to 8 dpi before decreasing to the original level at 8–16 dpi. Moreover, the SA content of Moneymaker gradually increased after inoculation, with a higher value than that of its control group at 0–3 dpi before decreasing to the minimum level observed at 12 dpi. In Cf*-16* tomato, the JA content rapidly increased after inoculation, peaking from 0 to 3 dpi, and then rapidly decreased between 4 and 21 dpi; however, the JA level was generally higher than that detected for the control groups of Cf*-16* tomato and Moneymaker after infection (from 2 to 16 dpi). In Moneymaker, the JA content increased after infection to a maximum at 3 dpi and then gradually decreased (Fig. 2b). Overall, the SA and JA levels in Moneymaker were greater after infection than those observed in its control group at 0–3 dpi. These results suggest that SA and JA levels rapidly increase during the early stages of infection and that these hormones play important roles in regulating the plant response to the pathogen and enhancing the defense of Cf*-16* tomato infected with *C. fulvum*.

### RNA sequencing and transcript identification

To obtain transcriptome profiles of Cf*-16* tomato and Moneymaker following *C. fulvum* infection, we performed RNA-Seq analysis at 4 and 8 dpi, with three biological replicates performed at each time point for each treatment. In this study, an average of ~ 6.87 Gb of clean data were generated for each sample using the BGISEQ-500 platform (Additional file 9: Figure S1, Additional file 1: Table S1). As shown in Additional file 1: Table S1, the quality scores of more than 98% of the reads were ≥ 20%, and more than 91% of the clean reads had quality scores of ≥30%. After the reads were filtered, 64.15–72.64 million clean reads were generated, and at least 93.29% of these reads were mapped to the tomato reference genome, among which more than 78.26% were aligned to unique locations. Ultimately, 18,514 novel transcripts were obtained, with 12,790 unknown splicing events in known genes, 2047 novel coding transcripts without any known features, and 3677 transcripts for long noncoding RNAs.

### DEGs in response to *C. fulvum*

DEGs from Cf*-16* tomato and Moneymaker in response to *C. fulvum* at 4 and 8 dpi were identified based on an adjusted *P*-value of ≤0.001 and a log_2_ fold change of ≥2. FPKM (fragments per kilobase of exon per million fragments mapped) values for all genes and the fold changes and adjusted *P*-values for DEGs are shown in Additional file 2: Table S2 and Additional file 3: Table S3, respectively.

In the control groups, 3298 and 2464 DEGs were observed between Cf*-16* tomato and Moneymaker at 4 and 8 dpi, respectively. Among the samples collected after infection with *C. fulvum* at 4 and 8 dpi, 2242 and 3095 DEGs were identified between Cf*-16* tomato and Moneymaker, respectively (Table 1). Compared with the respective control groups, 8526 DEGs were identified in Cf*-16* tomato (including 5110 up-regulated and 3416 down-regulated genes) at 4 dpi, 6938 in Moneymaker (including 4213 up-regulated and 2725 down-regulated genes) at 4 dpi, 3711 in Cf*-16* tomato (including 1609 up-regulated and 2102 down-regulated) at 8 dpi, and 2772 in Moneymaker (including 757 up-regulated and 2015 down-regulated genes) at 8 dpi.

**Table 1:** DEGs identified from different comparisons

DEG set	Total DEGs	Upregulated	Downregulated
CK_Cf_4dpi-vs-Cf_4dpi	8526	5110	3416
CK_Cf_8dpi-vs-Cf_8dpi	3711	1609	2102
CK_MM_4dpi-vs-MM_4dpi	6938	4213	2725
CK_MM_8dpi-vs-MM_8dpi	2772	757	2015
CK_MM_4dpi-vs-CK_Cf_4dpi	3298	1922	1376
CK_MM_8dpi-vs-CK_Cf_8dpi	2464	1361	1103
MM_4dpi-vs- Cf_4dpi	2242	1211	1031
MM_8dpi-vs- Cf_8dpi	3095	2043	1052

**Fig. 3:**
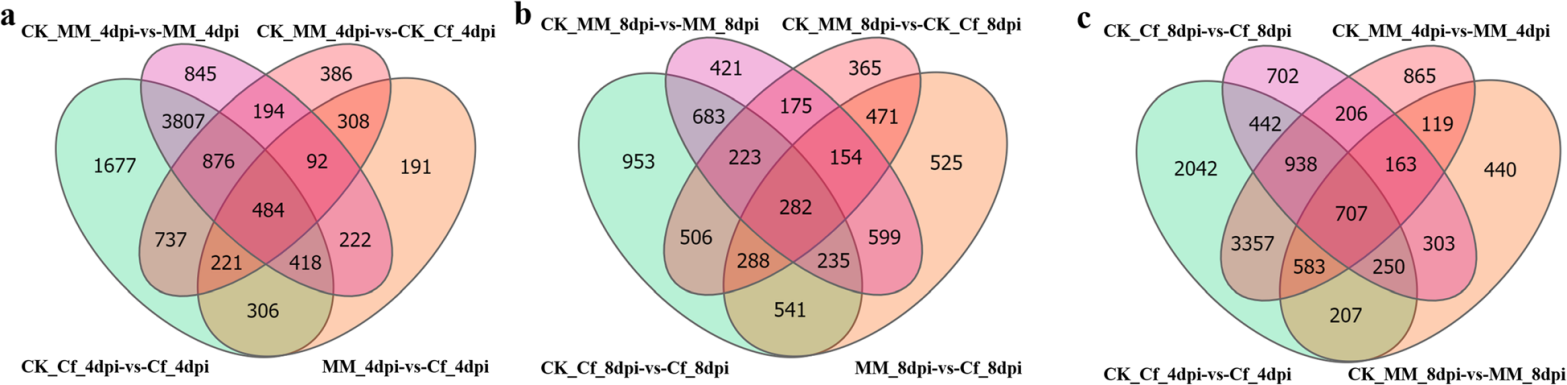
Venn diagrams showing DEGs in different comparisons post inoculation with *C. fulvum.* **a** Venn diagram of DEGs among the CK_Cf_4dpi-vs-Cf_4dpi, CK_MM_4dpi-vs-MM_4dpi, CK_MM_4dpi-vs-CK_Cf_4dpi and MM_4dpi-vs-Cf_4dpi comparisons. **b** Venn diagram of DEGs among the CK_Cf_8dpi-vs-Cf_8dpi, CK_MM_8dpi-vs-MM_8dpi, CK_MM_8dpi-vs-CK_Cf_8dpi and MM_8dpi-vs-Cf_8dpi comparisons. **c** Venn diagram of DEGs among the CK_Cf_4dpi-vs-Cf_4dpi, CK_MM_4dpi-vs-MM_4dpi, CK_Cf_8dpi-vs-Cf_8dpi and CK_MM_8dpi-vs-MM_8dpi comparisons. The numbers indicate the unique and common DEGs for the different comparisons. CK_Cf_4dpi, CK_Cf_8dpi, CK_MM_4dpi and CK_MM_8dpi: *Cf-16* tomato and Moneymaker were inoculated with water and collected at 4 and 8 dpi. Cf_4dpi, Cf_8dpi, MM_4dpi and MM_8dpi: *Cf-16* tomato and Moneymaker were inoculated with *C. fulvum* and collected at 4 and 8 dpi

Numerous DEGs were detected at 4 dpi in both cultivars (Table 1); however, we also noted a number of DEGs between the control groups for both cultivars (CK_MM_4dpi-vs-CK_Cf_4dpi and CK_MM_8dpi-vs-CK_Cf_8dpi). Furthermore, 707 DEGs were shared by the control and inoculation groups of Moneymaker and Cf*-16* tomato (Fig. 3c). Thus, some of the DEGs in each comparison may not be associated with leaf mold resistance. Notably, 306 DEGs overlapped between *Cf-16* tomato and Moneymaker only at 4 dpi (CK_Cf_4dpi-vs-Cf_4dpi and MM_4dpi-vs-Cf_4dpi) (Fig. 3a), whereas 541 DEGs overlapped only at 8 dpi (CK_Cf_8dpi-vs-Cf_8dpi and MM_8dpi-vs-Cf_8dpi) (Fig. 3b). Based on these stricter criteria, these DEGs are likely the most promising candidates involved in leaf mold resistance; accordingly, these DEGs were investigated further.

### GO and KEGG enrichment analyses of DEGs

**Fig. 4:**
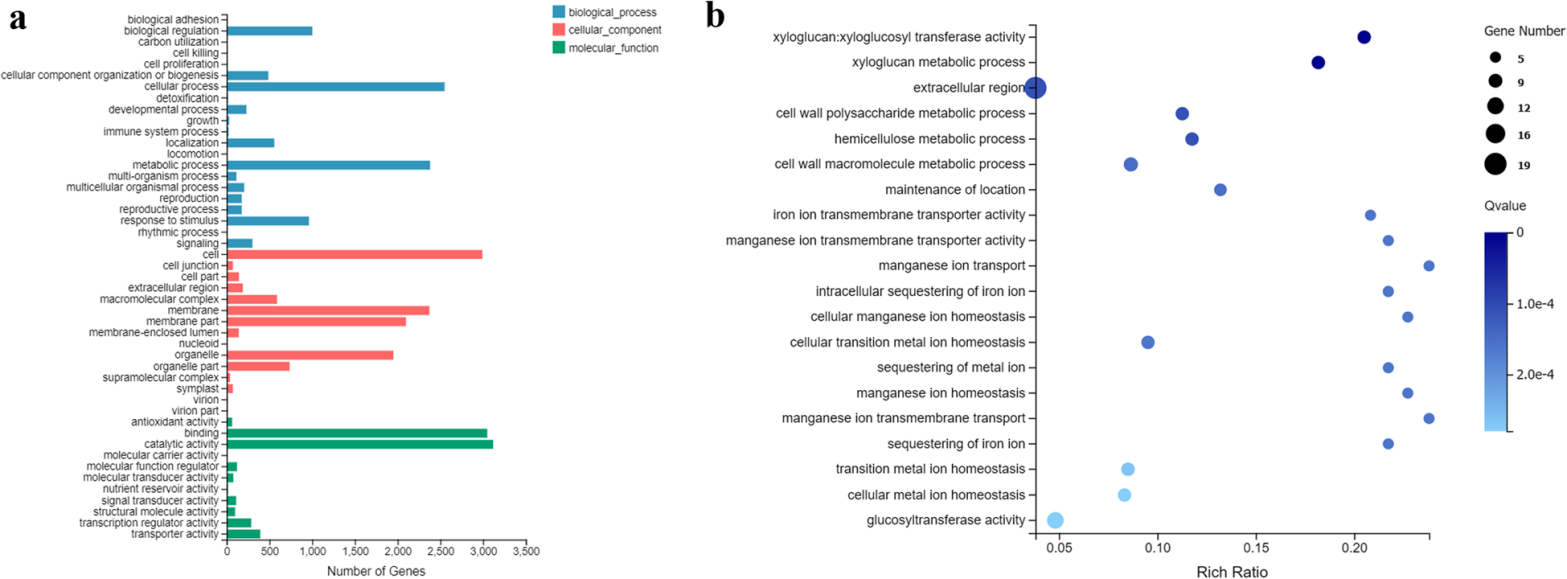
GO analysis of DEGs in response to *C. fulvum*. **a** GO classification analysis of the CK_Cf_4dpi-vs-Cf_4dpi comparison, **b** GO enrichment analysis of the top 200 DEGs from the CK_Cf_4dpi-vs-Cf_4dpi comparison

To determine the functions of DEGs involved in the response to *C. fulvum*, we performed GO (Gene Ontology) classification and KEGG (Kyoto Encyclopedia of Gene and Genomes) functional enrichment analyses using the Phyper function in R software [40]. For DEGs detected in Cf*-16* tomato, significant GO terms were primarily enriched in “biological regulation”, “cellular process”, “metabolic process” and “response to stimulus” in the biological process category, and these terms are associated with disease resistance. In the cellular component ontology, “cell”, “membrane”, “membrane part” and “organelle” were the most abundant categories that were specific to the resistant tomato cultivar. Genes involved in “binding”, “catalytic activity”, “transcription regulator activity” and “transporter activity” were enriched in the molecular function category. Notably, the terms “binding” and “catalytic activity” are known to play important roles in plant hormone signal transduction (Fig. 4a).

Subsequently, the top 200 DEGs between *Cf-16* tomato and its control group at 4 dpi (CK_Cf_4dpi-vs-Cf_4dpi) were selected for GO enrichment analysis to better understand the Cf*-16-*mediated resistance response to *C. fulvum*. As shown in Fig. 4b, the most highly enriched GO terms were those associated with the organization of the cell wall or the metabolism of its components, including “xyloglucan: xyloglucosyl transferase activity”, “xyloglucan metabolic process”, “cell wall polysaccharide metabolic process” and “hemicellulose metabolic process”. As the first barrier to invasion, the cell wall is the first obstacle for most pathogens [41, 42]. Therefore, DEGs associated with these significant terms may play important roles against *C. fulvum* infection in Cf*-16* tomato.

**Fig. 5:**
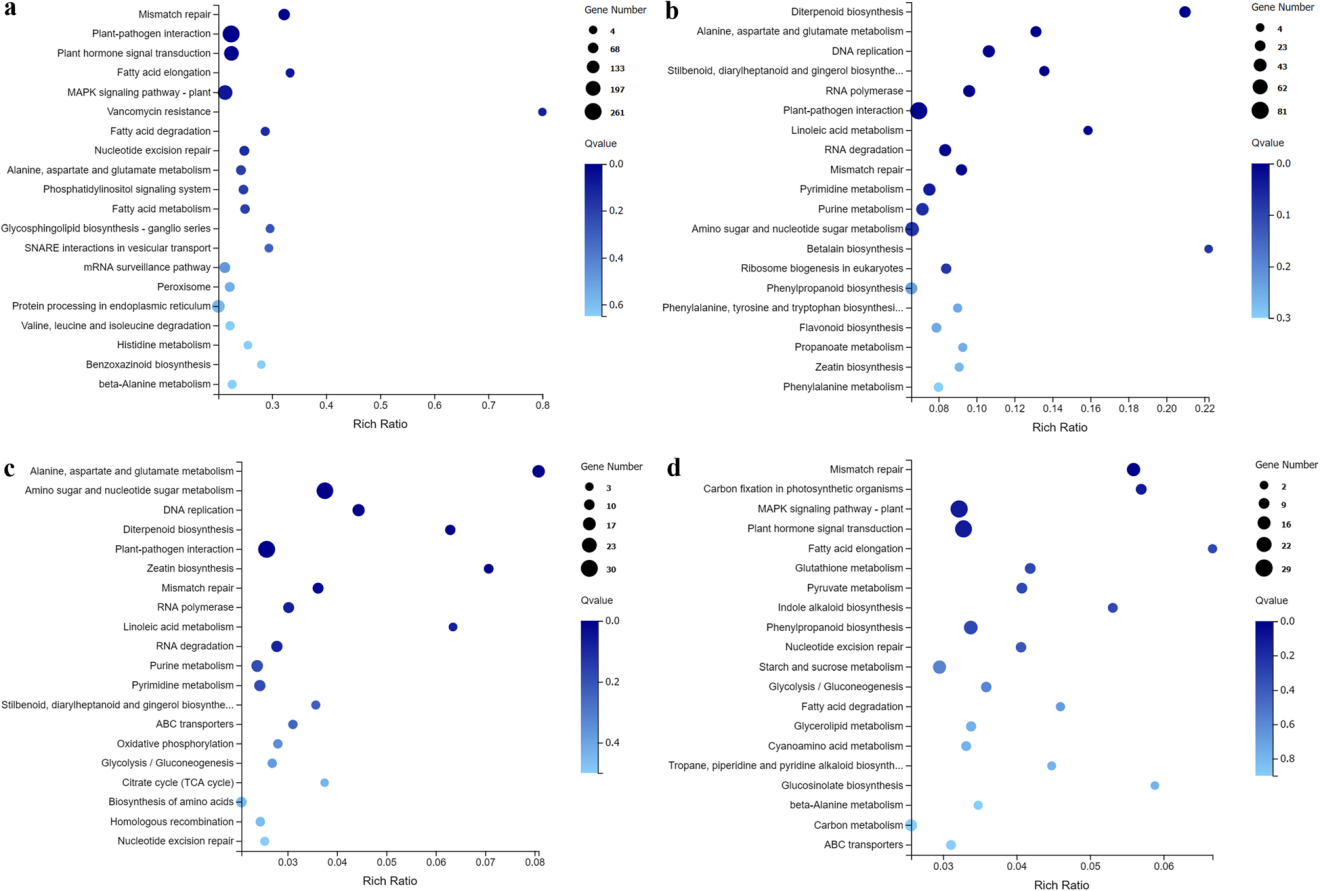
Scatter plot of KEGG pathway enrichment of DEGs. The rich ratio is the ratio of the DEG number to the background number in a particular pathway. The size of the dots represents the number of genes, and the color of the dots represents the range of the Q-value. **a** KEGG pathways based on upregulated DEGs in the CK_Cf_4dpi-vs-Cf_4dpi comparison. **b** KEGG pathways based on upregulated DEGs in the MM_4dpi-vs-Cf_4dpi comparison. **c** KEGG pathways based on 306 DEGs that overlapped only between the CK_Cf_4dpi-vs-Cf_4dpi and MM_4dpi-vs-Cf_4dpi comparisons according to the Venn diagram in Fig. 3a. **d** KEGG pathways based on 541 DEGs that overlapped only between the CK_Cf_8dpi-vs-Cf_8dpi and MM_8dpi-vs-Cf_8dpi comparisons according to the Venn diagram in Fig. 3b

KEGG pathway enrichment analysis was also performed to investigate the biological pathways underlying the incompatible interaction. As shown in Fig. 5a, the pathways “Plant hormone signal transduction” and “Plant-pathogen interaction” were significantly enriched (in the figure, the color of each dot indicates the Q-value, and the standard for significant enrichment is Q-value ≤0.01). In addition, “Fatty acid metabolism” and “Phosphatidylinositol signaling system” were found to be related to the Cf*-16* tomato response to *C. fulvum* infection. Furthermore, 34 disease-resistance genes (Additional file 6: Table S6) and 32 DEGs (Additional file 7: Table S7) were identified in the significantly enriched KEGG pathways “Plant-pathogen interaction” and “Plant hormone signal transduction”, respectively. In summary, the most highly enriched pathways, “Plant hormone signal transduction” and “Plant-pathogen interaction”, may be the major metabolic pathways involved in the Cf*-16*-mediated resistance response to *C. fulvum*.

Up-regulated genes involved in the “Plant-pathogen interaction” pathway were also significantly enriched between Moneymaker and Cf*-16* tomato (Fig. 5b, Additional file 8: Table S8). Thus, we performed KEGG pathway analysis for 306 and 541 DEGs that overlapped between Cf*-16* tomato and Moneymaker only at 4 and 8 dpi, respectively (Fig. 3a and b). Interestingly, the pathways “Plant-pathogen interaction” and “Plant hormone signal transduction” were significantly enriched (Fig. 5c and d). Some disease-resistance genes involved in “Plant-pathogen interaction” and six hormone-related genes involved in “Plant hormone signal transduction” are listed in Table 2 and Table 3, respectively. These DEGs may constitute the most promising candidates involved in leaf mold resistance. These results indicate once again that plant hormones play a key role in the *Cf-16* tomato response to *C. fulvum* infection.

**Table 2:** DEGs in the significantly enriched KEGG pathway “Plant-pathogen interaction” based on 306 common DEGs that overlapped only between the CK_Cf_4dpi-vs-Cf_4dpi and MM_4dpi-vs-Cf_4dpi comparisons according to the Venn diagram in Fig. 3a

Gene ID	Gene definition	Log_2_ Fold-change	
		CK_Cf_4dpi-vs-Cf_4dpi	MM_4dpi-vs-Cf_4dpi
101,246,100	putative ATPase	2.22	4.99
101,251,989	disease resistance protein	1.44	2.36
101,253,178	disease resistance protein RPM1	2.42	4.89
101,256,988	glucosamine---fructose-6-phosphate aminotransferase (isomerizing)	1.74	5.88
101,258,758	LRR receptor-like serine/threonine-protein kinase FLS2	1.77	1.60
101,263,364	putative ATPase	3.26	7.32
101,263,890	tubulin-folding cofactor B	1.26	2.13
109,118,687	disease resistance protein	2.34	1.61
109,120,689	disease resistance protein RPM1	2.94	5.15
109,121,092	LRR receptor-like serine/threonine-protein kinase FLS2	3.45	2.36
BGI_novel_G001085	5′-AMP-activated protein kinase, catalytic alpha subunit	1.80	2.34
BGI_novel_G001591	disease resistance protein RPM1	2.83	2.76

**Table 3:** DEGs in the significantly enriched KEGG pathway “Plant hormone signal transduction” based on 541 common DEGs that overlapped only between the CK_Cf_8dpi-vs-Cf_8dpi and MM_8dpi-vs-Cf_8dpi comparisons according to the Venn diagram in Fig. 3b

Gene ID	Gene definition	Log_2_ Fold-change	
		CK_Cf_8dpi-vs-Cf_8dpi	MM_8dpi-vs-Cf_8dpi
101,245,668	xyloglucan:xyloglucosyl transferase TCH4	4.16	2.63
101,251,578	aprataxin	3.19	2.20
101,262,506	arabidopsis histidine kinase 2/3/4 (cytokinin receptor)	1.32	2.13
101,263,609	disease resistance protein RPM1	1.70	1.54
101,264,326	SAUR family protein	1.75	3.76
104,649,076	auxin responsive GH3 gene family	4.19	2.62

### Analysis of metabolism and regulatory pathways

**Fig. 6:**
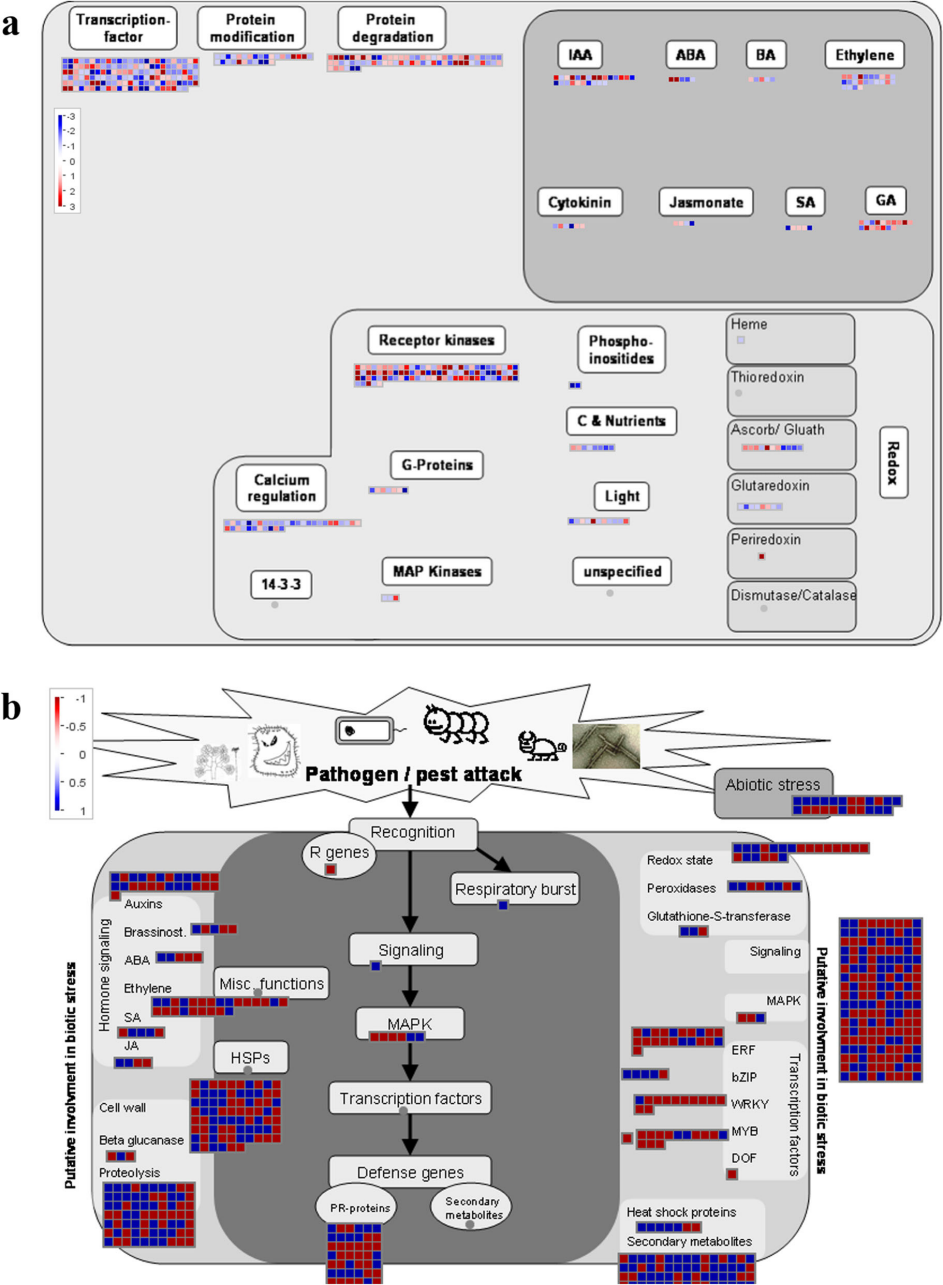
Regulatory overview produced by the MapMan tool. **a** biotic stress, **b** transcriptional changes in *Cf-16* tomato at 4 dpi (MM_4dpi-vs- Cf_4dpi). Each box represents a DEG; the red and blue colors indicate up- and downregulated DEGs, respectively. The scale bar displays log2-transformed fold changes

To obtain an overview of the regulatory pathways induced by *C. fulvum*, DEGs were visualized via MapMan analysis. According to the results (Fig. 6a), the majority of DEGs were upregulated and functionally enriched in transcription factors (TFs), including receptor kinases. Other pathways, including “calcium regulation” and “light”, were also upregulated or downregulated in response to *C. fulvum* infection. Most DEGs were related to hormones associated with the upregulation of IAA (indole-3-acetic acid), ABA (abscisic acid) and ethylene. In fact, the upregulated genes were classified as R genes, MAPKs, PR proteins, TFs and genes associated with the hormones ethylene, ABA, SA and JA, further supporting the importance of these pathways in the Cf*-*16-mediated resistance response to *C. fulvum* infection (Fig. 6b, Additional file 4: Table S4).

### Gene co-expression network analysis

**Fig. 7:**
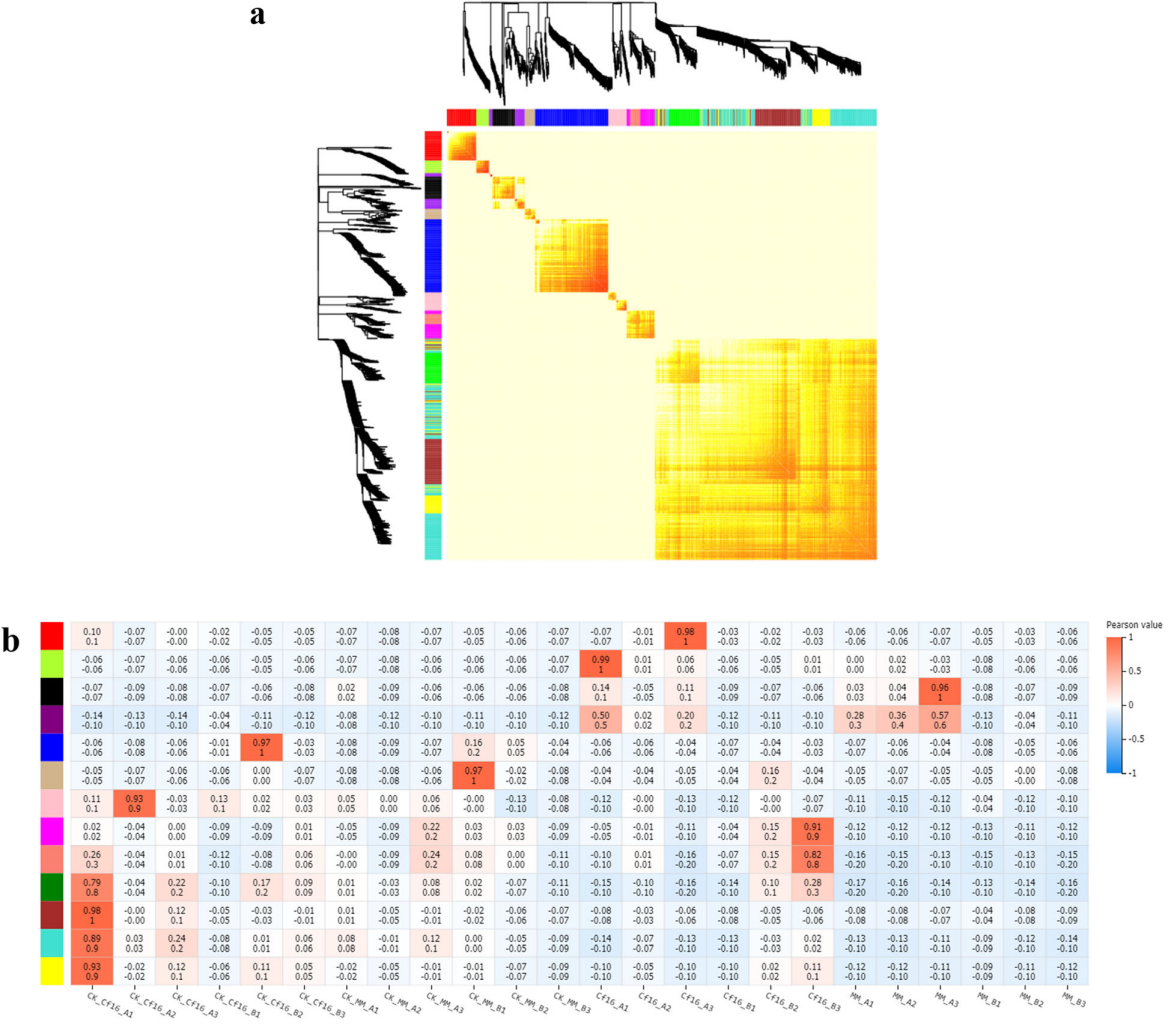
Gene co-expression network analysis by WGCNA. **a** Gene dendrogram colored according to correlations between gene expression levels. Different colors represent different gene modules and indicate coefficients of dissimilarity between genes. **b** Module-sample association. The abscissa represents the samples; the ordinate represents the modules. The numbers in each cell are the correlation coefficient (top) and *P*-value (bottom). The variation from blue (low) to orange (high) indicates the ranges of the DEGs

Weighted gene co-expression network analysis (WGCNA) is a common algorithm used in transcriptomic studies [43]. Thirteen different modules were obtained using a gene dendrogram colored according to correlations between gene expression levels (Fig. 7a). Among them, the genes in MEred and MEgreenyellow were highly expressed in Cf*-16* tomato at 4 dpi, whereas those in MEpurple exhibited relatively high expression in both Cf*-16* tomato and Moneymaker at 4 dpi (Fig. 7b). We performed KEGG analysis for these three modules. For the MEred module, pathways related to “Plant-pathogen interaction”, “Oxidative phosphorylation” and “Phenylalanine, tyrosine and tryptophan biosynthesis” were enriched; for MEgreenyellow, pathways related to “Pentose phosphate pathway”, “Flavonoid biosynthesis”, “Phenylpropanoid biosynthesis” and “Plant hormone signal transduction” were enriched (Additional file 10: Figure S2). Notably, some DEGs of the pathway “Plant-pathogen interaction” in the MEred module were also present in Additional file 8: Table S8 (DEGs in the significantly enriched KEGG pathway “Plant-pathogen interaction” between MM_4dpi-vs-Cf_4dpi and CK_Cf_4dpi-vs-Cf_4dpi). Therefore, these disease-resistance genes should be studied in greater depth in the future to elucidate their role in the Cf*-16*-mediated resistance response to *C. fulvum* infection in tomato.

### Validation of RNA-Seq data by qRT-PCR

**Fig. 8:**
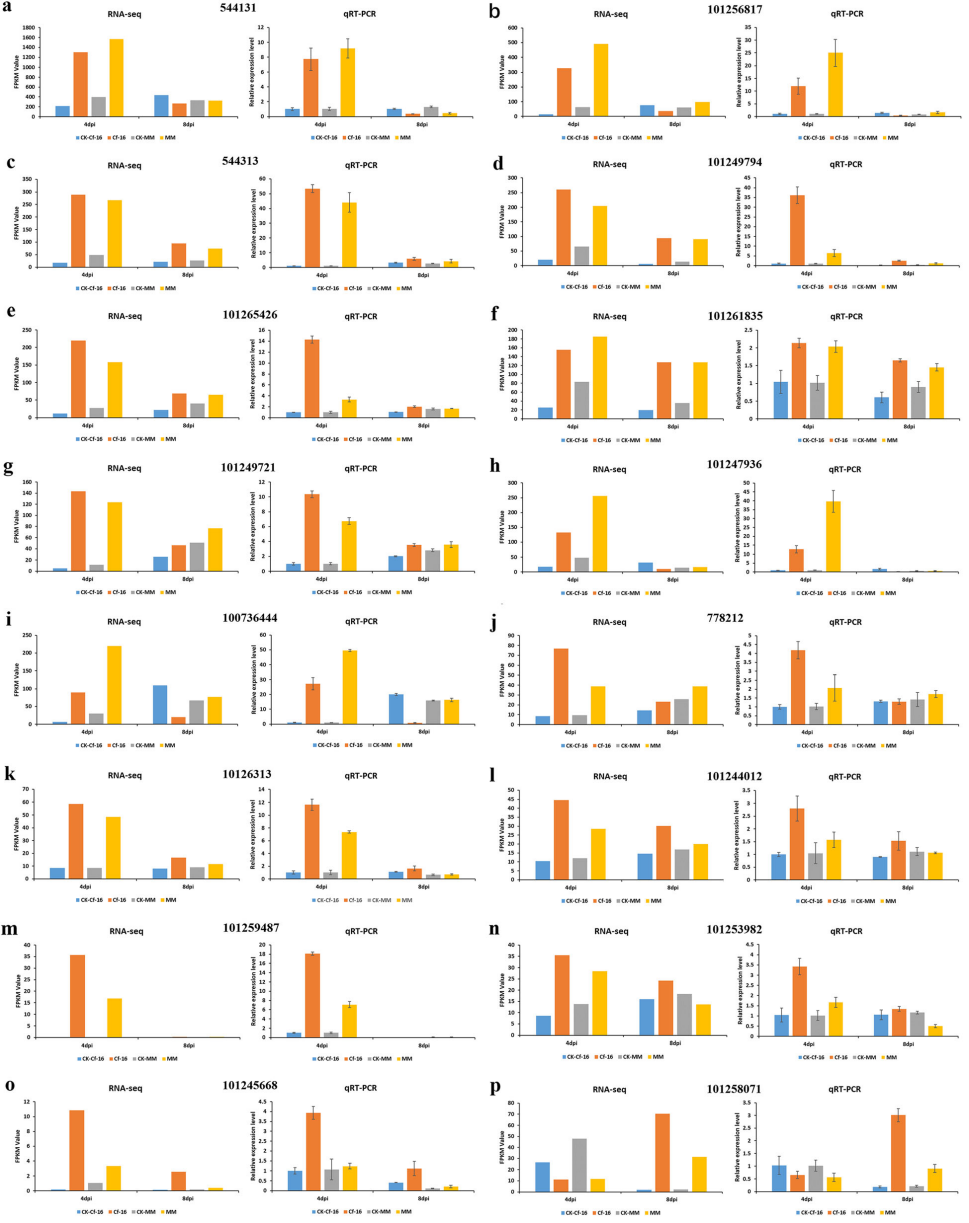
Comparative analysis of expression results between RNA-Seq and qRT-PCR for 16 DEGs

To verify the RNA-Seq data, 16 DEGs were chosen for qRT-PCR; three biological replicates were performed. These 16 genes were selected from significantly enriched KEGG pathways (such as “Plant hormone signal transduction”, “Plant-pathogen interaction” and “Metabolic pathways”). The expression data obtained by qRT-PCR were consistent with the RNA-Seq results, indicating a similar trend between the transcriptome and qRT-PCR datasets (Fig. 8). Among the 16 DEGs, a significantly up-regulated gene with ID 101247936 (Fig. 8h) was predicted to encode a jasmonate-ZIM-domain-containing protein in the “Plant hormone signal transduction” pathway, paralleling the JA response to *C. fulvum* infection (Fig. 2b). Similarly, the expression level of gene 100,736,444 (Fig. 8i), which encodes the disease-resistance protein RPM1, was increased at least 27-fold in Cf*-16* tomato. In addition, the expression levels of gene 101,259,487 (Fig. 8m), which encodes a peroxidase, and gene 101,256,817 (Fig. 8b), which encodes the calcium binding protein CML, were increased at least 11-fold.

## Discussion

In this study, we characterized the interaction between *C. fulvum* and Cf*-16* tomato or Moneymaker through microscopic observations. HR was observed in Cf*-16* tomato at 8 dpi, whereas in Moneymaker, hyphae emerged through the stomata and continued to increase in number and grow at 8 dpi (Fig. 1). Systemic defense response mediated by resistance genes was activated during the early stage of *C. fulvum* infection. This finding was consistent with previous studies of other Cf genes [37–39]. Based on comparative transcriptome analysis, we demonstrated the resistance response to *C. fulvum* in Cf*-16* tomato. In response to *C. fulvum* infection, drastic transcriptional changes occurred at 4 dpi in both tomato cultivars, although a number of DEGs were also detected between the respective control groups (Table 1). Because these results suggested that many of the DEGs in each comparison may not be linked to resistance, we screened resistance genes in Cf*-16* tomato more stringently by comparing the transcriptomes of the control groups. Significant GO terms primarily included “biological regulation”, “cellular process”, “metabolic process” and “response to stimulus” in the biological process category, and these terms are associated with disease resistance. Furthermore, KEGG enrichment analysis indicated that most of the DEGs were classified into “Plant hormone signal transduction” and “Plant-pathogen interaction”. In this preliminary comparison, more up-regulated DEGs were detected in Cf*-16* compared to Cf*-10* and Cf*-12* tomatoes during the early stage of *C. fulvum* infection [38, 39]. A comprehensive comparative analysis of Cf*-19,* Cf*-12*, Cf*-10* and Cf*-16* will provide important information for further exploration of the mechanism of Cf-gene-mediated resistance response to *C. fulvum* infection.

Plants have a series of defense mechanisms to respond to pathogen attack. PRRs are the first line of defense [44, 45]; these receptors recognize *C. fulvum* and activate a resistance response [46]. In our study, chitin elicitor receptor kinase 1 (CERK1; BGI_novel_G000519), a pattern recognition protein, was significantly up-regulated in *Cf-16* tomato at 4 dpi (Additional file 6: Table S6). This result was consistent with the study by Xue et al. (2017) on Cf*-12*. We will investigate whether increased expression of CERK1 is associated with the activation of chitin signaling and determine whether this increase affects the tomato-*C. fulvum* interaction process.

**Fig. 9:**
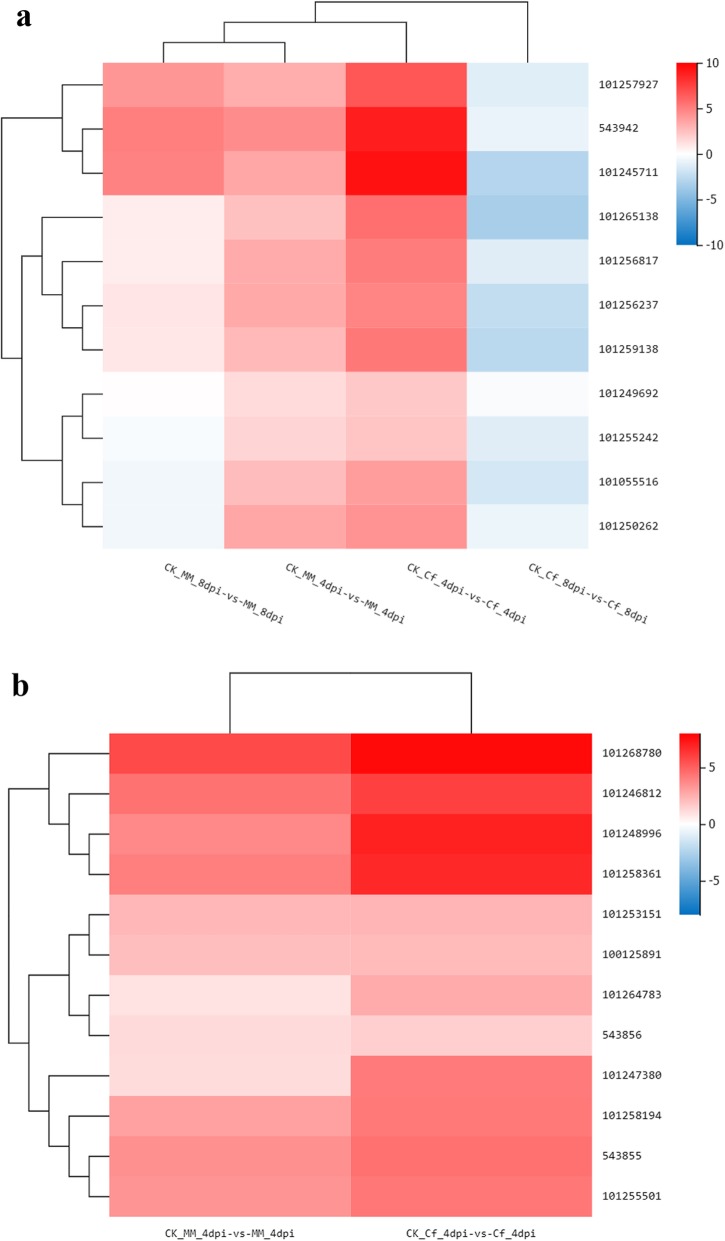
**a** Differentially expressed CML genes in Cf*-16* tomato and Moneymaker. **b** Differentially expressed WRKY genes in *Cf-16* tomato and Moneymaker at 4 dpi

After recognizing the infection, Cf*-16* tomato quickly activated a complex series of defense-associated signaling pathways. Ca^2+^ influx is considered to play a key role in the early downstream response of numerous PAMP sensing processes, resulting in local and systemic acquired resistance [47, 48]. Ca^2+^ activates calcium-dependent protein kinases (CDPKs), which play important roles in plant responses to both abiotic stress and pathogens [49, 50]. In our study, CDPKs (101,249,495, 101,055,527 and 101,255,379) were expressed at high levels in Cf*-16* tomato during the early stage of infection (Additional file 2: Table S2). This result was consistent with those of previous studies, suggesting that these genes play crucial roles in the Cf*-16-*mediated resistance response to *C. fulvum* infection [51]. In addition, the binding of Ca^2+^ to CML results in the production of nitric oxide (NO), which further promotes plant HR or autoimmune reactions [52]. Interestingly, the results of our study showed that 11 CML genes were significantly expressed at 4 dpi in Cf*-16* tomato compared with Moneymaker (Fig. 9a). In particular, the genes with IDs 543,942 and 101,245,711 were up-regulated approximately eight-fold in Cf*-16* tomato compared with Moneymaker. Based on these results, we propose that these CML genes are involved in the Cf*-16* tomato defense response against *C. fulvum*. Similarly, Ranty (2016) demonstrated that CML genes play crucial roles in plant responses to both abiotic stress and pathogens [53]. FLS2 recognizes flg22 and subsequently activates downstream signaling pathways that involve WRKY TFs to promote defense responses against bacterial and fungal pathogens and nematodes [54, 55]. In our study, 12 WRKY genes were specifically up-regulated at 4 dpi, as shown by the hierarchical clustering of DEGs in both tomato cultivars (Fig. 9b). Among them, the genes with IDs 101,268,780, 101,258,361, 101,248,996 and 101,246,812 were up-regulated more than six-fold in Cf*-16* tomato compared with Moneymaker. These results suggest that these WRKY genes may activate a series of downstream PR genes and thus play pivotal roles in the resistance response of Cf*-16* tomato to *C. fulvum*. Our results also showed that PR-1 genes (IDs 544,123 and 100,191,111) were significantly up-regulated in Cf*-16* tomato after inoculation (Additional file 6: Table S6). Overall, our results suggest that PRRs activate and promote the expression of downstream CDPKs, CMLs and WRKY TFs; induce the accumulation of reactive oxygen species; and cause the deposition of cystatin in the cell wall, thereby inducing PTI in Cf*-16* tomato.

During long-term evolutionary interactions with plants, several pathogens successfully cause ETS by producing a number of effectors. Simultaneously, plants have evolved R genes that recognize these effectors and function through highly specific interactions between effectors and their corresponding NB-LRR class receptors. The rice CC-NB-LRR protein Pi-to can directly interact with Avr factors, which the LRR domain is able to directly recognize the effector Avrpita of *Magnaporthe oryzae* and induce ETI [56]. It has also been demonstrated that the NBS-LRR protein from *Arabidopsis thaliana* RPM1 confers resistance to *Pseudomonas syringae*. RPM1 is also involved in the onset of HR [16, 57]. Consistent with previous studies, our results showed that genes encoding RPM1 (101,261,141, 100,736,444, 109,120,689, 101,253,178 and BGI_novel_G001591) were significantly up-regulated in Cf*-16* tomato at 4 dpi (Additional file 3: Table S3a). These genes may play a key role in the response of Cf*-16* tomato to *C. fulvum* infection. More importantly, the genes with IDs 109,120,689, 101,253,178 and BGI_novel_G001591 were identified as DEGs that overlapped between Cf*-16* tomato and Moneymaker only at 4 dpi (Table 2). These genes may be the most promising candidate genes involved in leaf mold resistance.

Phytohormones are known to be important in the regulation of defense responses in plants [58–60]. SA, a crucial regulator of plant-pathogen interactions, induces HR and systemic acquired resistance [61]. In our study, 32 DEGs were identified in the significantly enriched KEGG pathway “Plant hormone signal transduction” (Additional file 7: Table S7). Interestingly, TGA (104,645,854, 101,250,172 and 101,253,982) and PR-1 (544123), which are involved in the SA signaling pathway, were significantly up-regulated in Cf*-16* tomato after *C. fulvum* inoculation. Importantly, the expression of PR-1 (544123) was significantly higher in Cf*-16* tomato than in Moneymaker at 4 and 8 dpi, suggesting that PR-1 may have a significant function in the response of Cf*-16* tomato to *C. fulvum* infection. This result is consistent with previous studies [62]. We also showed that JAZ (jasmonate-zim-domain) genes (101,247,936 and 100,134,911), which encode major proteins in JA signaling, were up-regulated in Cf*-16* tomato at 4 dpi. This finding is consistent with the previously measured change in JA content after infection. Additionally, SAUR family proteins (BGI_novel_G000650, BGI_novel_G001679, 101,255,313, 101,257,321, 104,648,957 and 101,264,326) and PP2Cs (101,249,794 and 101,261,835) in the KEGG pathway “Plant hormone signal transduction” (Additional file 7: Table S7) were identified in the present study, suggesting that SAUR family proteins and PP2Cs also play roles in the resistance of Cf*-16* tomato to *C. fulvum*. In particular, the SAUR family protein with ID 101264326 was identified among the DEGs that overlapped between Cf*-16* tomato and Moneymaker only at 8 dpi (Table 3). Elucidation of the function of this protein in the response of Cf*-16* tomato to *C. fulvum* is needed. Overall, discrepancies among different studies suggest that the specific hormones involved may vary and behave differently in different tomato-*C. fulvum* interactions under different conditions and at different time points. Therefore, it is important to explore crosstalk between SA and JA signaling in the activation of Cf*-16*-mediated defense systems against *C. fulvum* attack and close interactions with other Cf genes.

## Conclusions

This study analyzed the first comprehensive transcriptome of the Cf*-16* resistant tomato cv. Ontario7816 and susceptible tomato cv. Moneymaker and explored interactions between Cf*-16* tomato and *C. fulvum*. Some DEGs related to disease resistance were identified and predicted to be associated with the plant innate immune response, Ca^2+^ channels and plant hormone signal transduction pathways. These results contribute to our understanding of the potential mechanism by which Cf*-16* tomato combats *C. fulvum* infection and will facilitate the fine mapping and cloning of the Cf*-16* gene in the future.

## Methods

### Plant materials and *C. fulvum* inoculation

Two tomato cultivars, the resistant cultivar Ontario7816, including the Cf*-16* gene (kindly provided by the Institute of Vegetables and Flowers, Chinese Academy of Agricultural Science) [23], and the susceptible cultivar Moneymaker, which lacks *C. fulvum*-resistance genes (kindly provided by the Tomato Genetic Resource Center, LA2706), were used in this study. Tomato seeds were sown in pots filled with soil and grown under controlled conditions (16 h light, 25 °C and 95% ambient humidity) in a greenhouse at the Horticultural Station of Northeast Agricultural University (Harbin, China). At the four- to six-leaf stage, the abaxial leaf surfaces of 40 plants per line were sprayed with a suspension of *C. fulvum* (race 1.2.3.4) at 1 × 10^7^ sporangia per milliliter [63]. Mock-treated plants of each line were sprayed with sterilized water under the same conditions. All plants were maintained at 25 °C with 95% relative humidity.

### Microscopic observation of *C. fulvum* in Cf*-16* tomato

To assess the process of Cf*-16*-mediated HR and the key time points involved in the resistance mechanism, the lactophenol trypan blue staining method was performed according to Franco’s approach [64]. Leaf samples from the resistant and susceptible lines were harvested at 0–21 dpi, immediately stained, clarified overnight in chloral hydrate solution (2.5 mg/ml) [65], and examined using an Olympus SZX10 dissecting microscope (Olympus, Japan).

### Endogenous JA and SA levels

Leaf samples from the inoculation and control groups of the resistant and susceptible cultivars were harvested at 0, 2, 3, 4, 8, 12, 16 and 21 dpi. Endogenous SA and JA were extracted from the leaves using the modified method of Llugany et al. (2013) [66]; levels were measured via HPLC-MS/MS using an AB SCIEX Triple TOF5600^+^ mass spectrometer (SCIEX, USA) [67].

### RNA extraction, cDNA library construction and sequencing

Total RNA was obtained from each group at 4 and 8 dpi, for a total of 24 samples, and used for RNA-Seq and qRT-PCR analyses. Total RNA was extracted from three biological replicates for each group with three plants using the RNAprep Pure Plant Kit (ThermoFisher, USA) and then used for qRT-PCR [68, 69]; quantified RNA samples were used for cDNA library construction. Library preparation and sequencing were conducted by BGI Tech (Shenzhen, China). The libraries were generated using NEBNext® Ultra™ RNA Library Prep Kit for Illumina R (NEB, USA) and sequenced using a BGISEQ-500, with 150-bp paired-end reads generated. The raw sequencing data were deposited in NCBI Sequence Read Archive under the accession number GSE133678 (https://www.ncbi.nlm.nih.gov/geo/query/acc.cgi?acc=GSE133678).

### Sequencing read mapping and identification of DEGs

Raw reads in FASTQ format were generated by base calling, statistically analyzed using SOAPnuke v1.4.0 and filtered using Trimmomatic v0.36 [70]. Clean reads were obtained by removing reads with adapters, reads containing more than 5% poly-N (where N represents unknown bases), and low-quality reads (with a mass value less than 10 and proportion of total number of bases in the reads greater than 20%).

The clean reads were aligned to the *S. lycopersicum* reference genome sequence (NCBI_GCF_000188115.3_SL2.50) using HISATv2.1.0 [71]. Gene expression levels were quantified with the FPKM method using RSEMv1.2.8 [40]. DEGs were detected using DEGseq methods based on the Poisson distribution [72]. Genes with an adjusted *P*-value of ≤0.001 and a log_2_ fold change of ≥2 were defined as differentially expressed [73].

### Functional annotation and enrichment pathway analyses of DEGs

GO and KEGG pathway enrichment analyses of DEGs were performed using the Phyper function in R software; GO terms and KEGG pathways with an adjusted P-value of ≤0.01 were regarded as significantly enriched. For a graphical overview, DEGs were mapped to various metabolic and regulatory pathways (bins) using the MapMan tool. The colored boxes in each bin represent the log_2_-transformed fold change values of the DEGs.

### Gene co-expression network analysis

Gene co-expression network analysis was performed using the WGCNA package v1.48. Gene dendrograms were constructed with colors based on the correlations between the expression levels of genes and used to build clustering trees and to divide modules. In addition, the correlation between modules and samples was analyzed using WGCNA.

### qRT-PCR analysis

Sixteen DEGs were validated using qRT-PCR to verify the expression profiles obtained by RNA-Seq. qRT-PCR was performed using AceQ® qPCR SYBR® Green Master Mix (Vazyme, USA) and a qTOWER^3^G Detection System (Analytik Jena, Germany). Each sample was replicated three times, and data analysis was performed using the 2^-△△CT^ method [74]. The gene *EFα1* was used as a reference control for normalization (R: 5′-CCACCAATCTTGTACACATCC-3′, S: 5′-AGACCACCAAGTACTACTGCAC-3′) (Additional file 5: Table S5).

## Supplementary information

**Additional file 1: Table S1.** Summary of RNA-Seq data.

**Additional file 2: Table S2.** All FPKM values for every gene.

**Additional file 3: Table S3.** All fold changes and *P*-values for DEGs between the different groups.

**Additional file 4: Table S4.** DEGs between Moneymaker and Cf*-16* tomato at 4 dpi, based on MapMan analysis.

**Additional file 5: Table S5.** Primers used for qPCR.

**Additional file 6: Table S6.** Up-regulated DEGs in the significantly enriched KEGG pathway “Plant-pathogen interaction” in Cf*-16* tomato and Moneymaker at 4 dpi.

**Additional file 7: Table S7.** Up-regulated DEGs in the significantly enriched KEGG pathway “Plant hormone signal transduction” in Cf*-16* tomato and Moneymaker at 4 dpi.

**Additional file 8: Table S8.** Common DEGs in the significantly enriched KEGG pathway “Plant-pathogen interaction” between the MM_4dpi-vs-Cf_4dpi and CK_Cf_4dpi-vs-Cf_4dpi comparisons.

**Additional file 9: Figure S1.** Classification of raw reads from different samples.

**Additional file 10: Figure S2.** Scatter plot of KEGG pathway enrichment of the three modules from WGCNA.

## Data Availability

All the data pertaining to the present study have been included in the tables and figures of the manuscript. The raw sequencing data were deposited in NCBI Sequence Read Archive under the accession number GSE133678 (https://www.ncbi.nlm.nih.gov/geo/query/acc.cgi?acc=GSE133678).

## Abbreviations

ABA: Abscisic acid
cDNA-AFLP: cDNA-amplified fragment length polymorphism
CDPK: Calcium-dependent protein kinase
CERK1: Chitin elicitor receptor kinase 1
CML: Calcium binding protein
CNCG: Plant cyclic nucleotide-gated ion channel
DEG: Differentially expressed gene
dpi: Day post inoculation
ETI: Effector-triggered immunity
ETS: Effector-triggered susceptibility response
FPKM: Fragments per kilobase of exon per million fragments mapped
GO: Gene Ontology
HPLC-MS/MS: High-performance liquid chromatography-tandem mass spectrometry
HR: Hypersensitive response
IAA: Indole-3-acetic acid
JA: Jasmonic acid
JAZ: Jasmonate-zim-domain gene
KEGG: Kyoto Encyclopedia of Genes and Genomes
NB-LRR: Nucleotide-binding site and leucine-rich repeat
PAMP: Pathogen-associated molecular pattern
PRR: Pattern recognition receptor
PTI: PAMP-triggered immunity
qRT-PCR: Real-time quantitative polymerase chain reaction
SA: Salicylic acid
TF: Transcription factor
WGCNA: Weighted gene coexpression network analysis
