# Supplementary material for: Comparative transcriptome analysis reveals the response mechanism of Cf-16-mediated resistance to Cladosporium fulvum infection in tomato
Source: BMC Plant Biol. 2020 Jan 20;20:33. doi: 10.1186/s12870-020-2245-5 (PMC6971981; doi:10.1186/s12870-020-2245-5)
Supplement: Supplementary file 6 — Additional file 6: Table S6. Up-regulated DEGs in the significantly enriched KEGG pathway “Plant-pathogen interaction” in Cf-16 tomato and Moneymaker at 4 dpi. [file 12870_2020_2245_MOESM6_ESM.docx]

**Table S6** Upregulated DEGs in the significantly enriched KEGG pathway “Plant-pathogen interaction” in Ontario7816 and Moneymaker at 4 dpi.

| Gene ID | Gene definition | Log_2_ Fold-change | |
| --- | --- | --- | --- |
|  |  | CK_Cf_4dpi-vs-Cf_4dpi | CK_MM_4dpi-vs-MM_4dpi |
| 101055527 | calcium-dependent protein kinase | 3.48 | 2.95 |
| 101245711 | calcium-binding protein CML | 8.41 | 3.13 |
| 544043 | pathogenesis-related genes transcriptional activator PTI6 | 1.88 | 1.17 |
| 101251749 | calmodulin | 2.65 | 1.69 |
| 544123 | pathogenesis-related protein 1 | 4.63 | 2.38 |
| 543942 | calcium-binding protein CML | 7.99 | 4.02 |
| 101268780 | WRKY transcription factor 1 | 7.73 | 5.68 |
| 101248996 | WRKY transcription factor 33 | 7.01 | 3.74 |
| 101258361 | WRKY transcription factor 2 | 6.75 | 4.04 |
| 101265539 | interleukin-1 receptor-associated kinase 1 | 6.14 | 2.38 |
| 101257927 | calcium-binding protein CML | 5.97 | 2.89 |
| 104644839 | leucine-rich repeat protein SHOC2 | 5.86 | 3.68 |
| 543900 | EIX receptor 1/2 | 5.20 | 4.44 |
| 101265138 | calcium-binding protein CML | 5.10 | 2.33 |
| 101249495 | calcium-dependent protein kinase | 5.03 | 2.85 |
| 101259138 | calcium-binding protein CML | 4.78 | 2.47 |
| 104644838 | leucine-rich repeat protein SHOC2 | 4.54 | 3.57 |
| 101255316 | EIX receptor 1/2 | 4.38 | 3.00 |
| 104645857 | LRR receptor-like serine/threonine-protein kinase FLS2 | 4.35 | 3.28 |
| 101250219 | cyclic nucleotide gated channel, plant | 4.09 | 3.41 |
| 101261141 | disease resistance protein RPM1 | 4.09 | 2.58 |
| 100736444 | disease resistance protein RPM1 | 3.86 | 2.73 |
| 100191111 | pathogenesis-related protein 1 | 3.38 | 2.70 |
| 101257064 | LRR receptor-like serine/threonine-protein kinase FLS2 | 3.33 | 2.45 |
| 101257866 | pathogen-induced protein kinase | 2.89 | 1.92 |
| 101254274 | pathogenesis-related genes transcriptional activator PTI6 | 2.25 | 2.06 |
| 101246812 | WRKY transcription factor 33 | 5.99 | 4.44 |
| 101251005 | interleukin-1 receptor-associated kinase 4 | 5.01 | 3.38 |
| 101256817 | calcium-binding protein CML | 4.62 | 2.96 |
| 101255379 | calcium-dependent protein kinase | 2.62 | 1.47 |
| BGI_novel_G000519 | chitin elicitor receptor kinase 1 | 2.62 | 1.52 |
| 101253178 | disease resistance protein RPM1 | 2.42 | / |
| 109120689 | disease resistance protein RPM1 | 2.94 | / |
| BGI_novel_G001591 | disease resistance protein RPM1 | 2.83 | / |
