# Supplementary material for: Comparative transcriptome analysis reveals the response mechanism of Cf-16-mediated resistance to Cladosporium fulvum infection in tomato
Source: BMC Plant Biol. 2020 Jan 20;20:33. doi: 10.1186/s12870-020-2245-5 (PMC6971981; doi:10.1186/s12870-020-2245-5)
Supplement: Supplementary file 7 — Additional file 7: Table S7. Up-regulated DEGs in the significantly enriched KEGG pathway “Plant hormone signal transduction” in Cf-16 tomato and Moneymaker at 4 dpi. [file 12870_2020_2245_MOESM7_ESM.docx]

**Table S7** Upregulated DEGs in the significantly enriched KEGG pathway “Plant hormone signal transduction” in Ontario7816 and Moneymaker at 4 dpi.

| Gene ID | Gene definition | Log_2_ Fold-change | |
| --- | --- | --- | --- |
|  |  | CK_Cf_4dpi-vs-Cf_4dpi | CK_MM_4dpi-vs-MM_4dpi |
| 101249794 | protein phosphatase 2C | 3.71 | 1.64 |
| 101261835 | protein phosphatase 2C | 2.55 | 1.13 |
| 101247936 | jasmonate ZIM domain-containing protein | 2.90 | 2.44 |
| 100037510 | serine/threonine-protein kinase SRK2 | 1.98 | 1.82 |
| 101253982 | transcription factor TGA | 2.04 | 1.04 |
| 101245668 | xyloglucan: xyloglucosyl transferase TCH4 | 5.83 | 1.69 |
| 100037501 | ATP-dependent RNA helicase DDX47/RRP3 | 2.50 | 1.72 |
| 100134911 | jasmonate ZIM domain-containing protein | 3.14 | 3.03 |
| 100191111 | pathogenesis-related protein 1 | 3.38 | 2.70 |
| 101246381 | abscisic acid receptor PYR/PYL family | 3.79 | 2.25 |
| 101247146 | ubiquitin carboxyl-terminal hydrolase 7 | 4.58 | 3.14 |
| 101248216 | protein brassinosteroid insensitive 1 | 2.21 | 1.34 |
| 101255313 | SAUR family protein | 2.10 | 1.17 |
| 101257321 | SAUR family protein | 2.65 | 1.82 |
| 101258345 | xyloglucan:xyloglucosyl transferase TCH4 | 8.84 | 6.91 |
| 101258926 | xyloglucan:xyloglucosyl transferase TCH4 | 7.73 | 6.39 |
| 101262480 | gibberellin receptor GID1 | 3.37 | 2.18 |
| 104645854 | transcription factor TGA | 4.87 | 1.20 |
| 104648957 | SAUR family protein | 3.80 | 3.25 |
| 543518 | ethylene-insensitive protein 3 | 1.80 | 1.60 |
| 544101 | xyloglucan: xyloglucosyl transferase | 4.92 | 3.70 |
| 544123 | pathogenesis-related protein 1 | 4.63 | 2.38 |
| BGI_novel_G000650 | SAUR family protein | 3.73 | 1.60 |
| BGI_novel_G001679 | SAUR family protein | 5.12 | 2.98 |
| BGI_novel_G001690 | ethylene receptor | 4.51 | 2.80 |
| 544134 | abscisic acid receptor PYR/PYL family | 4.42 | 1.42 |
| 101267321 | DELLA protein | 2.69 | 1.50 |
| 109118687 | disease resistance protein | 2.34 | 0.84 |
| 101258707 | gibberellin receptor GID1 | 2.17 | 0.93 |
| 101265119 | gibberellin 2-oxidase | 2.01 | 0.17 |
| 101250172 | transcription factor TGA | 4.42 | / |
| 101253982 | transcription factor TGA | 2.04 | 1.04 |
