# Supplementary material for: Comparative transcriptome analysis reveals the response mechanism of Cf-16-mediated resistance to Cladosporium fulvum infection in tomato
Source: BMC Plant Biol. 2020 Jan 20;20:33. doi: 10.1186/s12870-020-2245-5 (PMC6971981; doi:10.1186/s12870-020-2245-5)
Supplement: Supplementary file 8 — Additional file 8: Table S8. Common DEGs in the significantly enriched KEGG pathway “Plant-pathogen interaction” between the MM_4dpi-vs-Cf_4dpi and CK_Cf_4dpi-vs-Cf_4dpi comparisons. [file 12870_2020_2245_MOESM8_ESM.docx]

**Table S8** Common DEGs in the significantly enriched KEGG pathway “Plant-pathogen

interaction” between MM_4dpi-vs-Cf_4dpi and CK_Cf_4dpi-vs- Cf_4dpi.

| Gene ID | Gene definition | Log_2_ Fold-change | |
| --- | --- | --- | --- |
|  |  | CK_Cf_4dpi-vs-Cf_4dpi | MM_4dpi-vs-Cf_4dpi |
| 101246100 | putative ATPase | 2.22 | 4.99 |
| 101251989 | disease resistance protein | 1.44 | 2.36 |
| 101253178 | disease resistance protein RPM1 | 2.42 | 4.89 |
| 101250668 | putative ATPase | 1.83 | 6.13 |
| 101251339 | putative ATPase | 3.41 | 6.78 |
| 101252423 | LRR receptor-like serine/threonine-protein kinase FLS2 | 1.06 | 11.01 |
| 101253568 | disease resistance protein RPS2 | 1.61 | 3.28 |
| 101256988 | glucosamine---fructose-6-phosphate aminotransferase (isomerizing) | 1.74 | 5.88 |
| 101263364 | putative ATPase | 3.26 | 7.32 |
| 101263890 | tubulin-folding cofactor B | 1.26 | 2.13 |
| 101265119 | gibberellin 2-oxidase | 2.01 | 3.45 |
| 104646013 | glucosamine---fructose-6-phosphate aminotransferase (isomerizing) | 2.18 | 7.40 |
| 104646849 | glucosamine---fructose-6-phosphate aminotransferase (isomerizing) | 2.79 | 8.17 |
| 104648490 | putative ATPase | 2.09 | 4.23 |
| 104649101 | glucosamine---fructose-6-phosphate aminotransferase (isomerizing) | 2.55 | 7.17 |
| 109118687 | disease resistance protein | 2.34 | 1.61 |
| 109119483 | oligoribonuclease | 2.09 | 3.22 |
| 109120295 | vacuolar protein sorting-associated protein 13A/C | 1.99 | 4.58 |
| 109120689 | disease resistance protein RPM1 | 2.94 | 5.15 |
| 109121092 | LRR receptor-like serine/threonine-protein kinase FLS2 | 3.45 | 2.36 |
| 109121288 | gibberellin 2-oxidase | 1.81 | 2.42 |
| BGI_novel_G000518 | chitin elicitor receptor kinase 1 | 5.86 | 2.13 |
| BGI_novel_G001085 | 5'-AMP-activated protein kinase, catalytic alpha subunit | 1.80 | 2.34 |
| BGI_novel_G001112 | lipoyl synthase | 2.09 | 2.57 |
| BGI_novel_G001591 | disease resistance protein RPM1 | 2.83 | 2.76 |
