# Supplementary figures and images for: Comparative transcriptome analysis reveals the response mechanism of Cf-16-mediated resistance to Cladosporium fulvum infection in tomato
Source: BMC Plant Biol. 2020 Jan 20;20:33. doi: 10.1186/s12870-020-2245-5 (PMC6971981; doi:10.1186/s12870-020-2245-5)

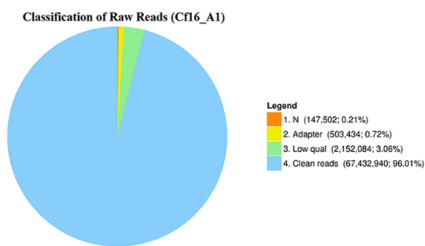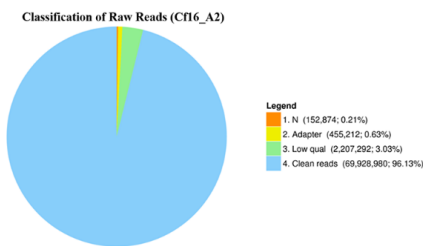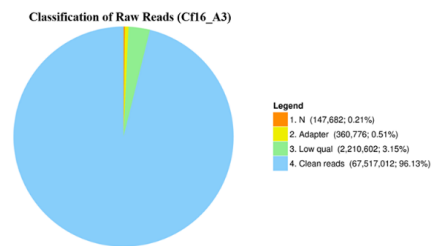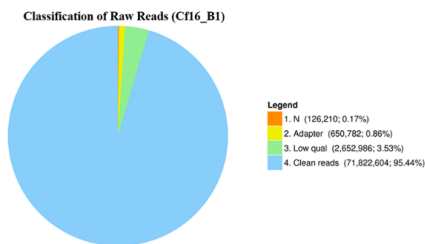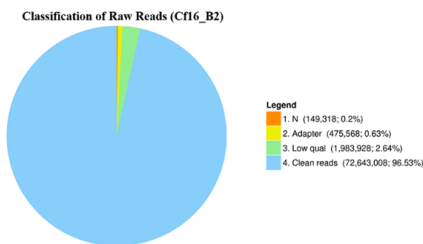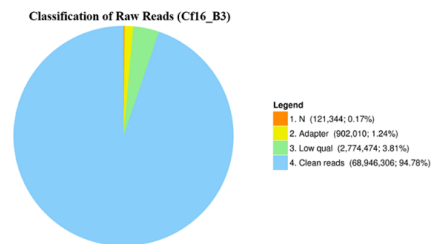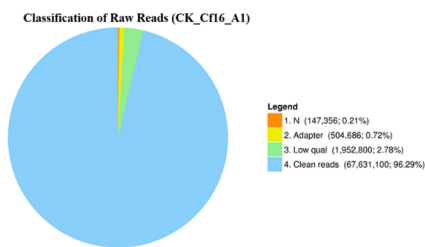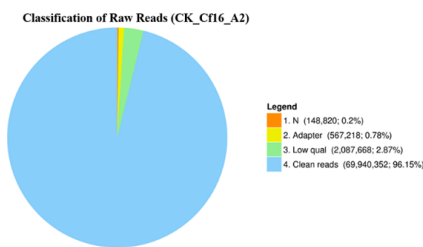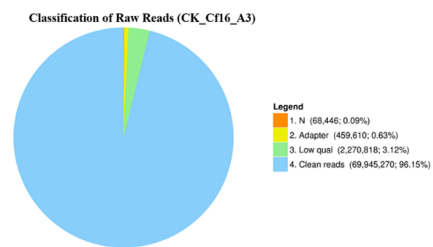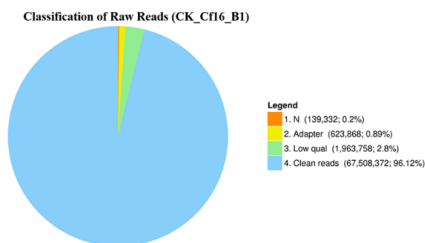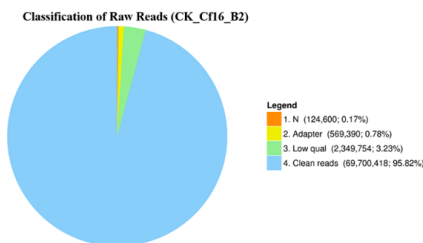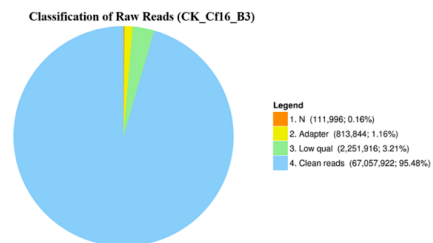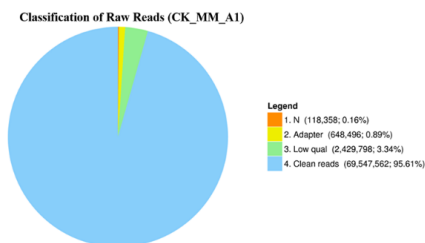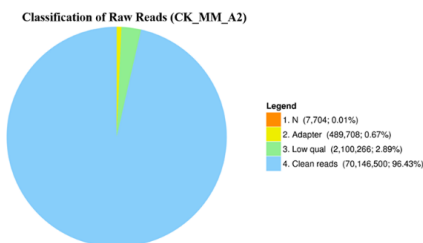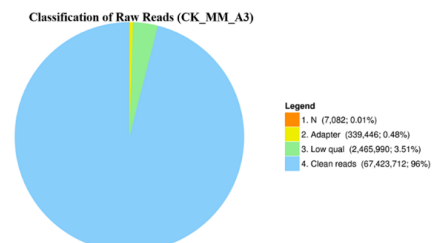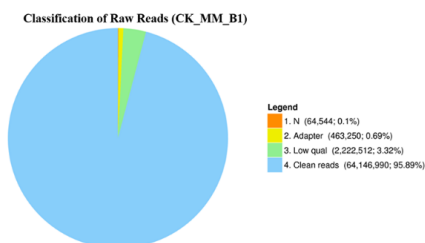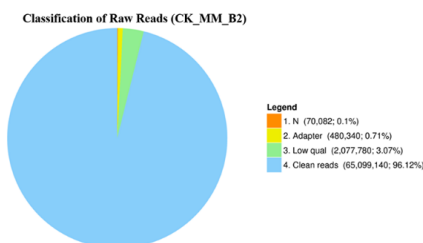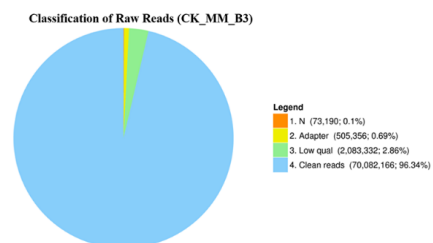

**Fig. S1 Classification of raw reads of different samples.**

Supplement: Supplementary file 9 — Additional file 9: Figure S1. Classification of raw reads from different samples. [file 12870_2020_2245_MOESM9_ESM.pdf]
