# Supplementary material for: Comparative transcriptome analysis reveals the response mechanism of Cf-16-mediated resistance to Cladosporium fulvum infection in tomato
Source: BMC Plant Biol. 2020 Jan 20;20:33. doi: 10.1186/s12870-020-2245-5 (PMC6971981; doi:10.1186/s12870-020-2245-5)
Supplement: Supplementary file 10 — Additional file 10: Figure S2. Scatter plot of KEGG pathway enrichment of the three modules from WGCNA. [file 12870_2020_2245_MOESM10_ESM.pdf]

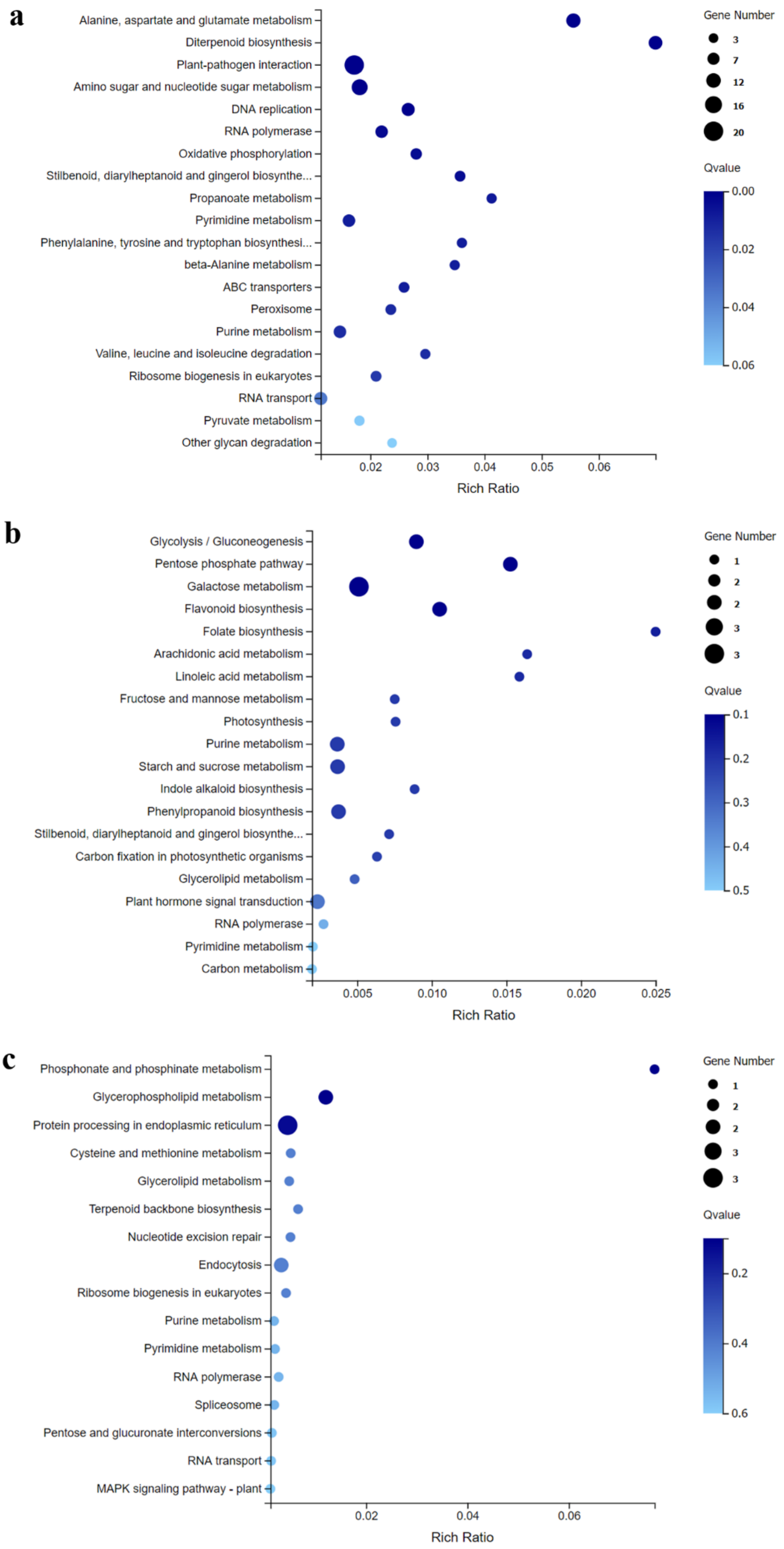
a
Alanine, aspartate and glutamate metabolism
Diterpenoid biosynthesis
Plant-pathogen interaction
Amino sugar and nucleotide sugar metabolism
DNA replication
RNA polymerase
Oxidative phosphorylation
Stilbenoid, diarylheptanoid and gingerol biosynthe...
Propanoate metabolism
Pyrimidine metabolism
Phenylalanine, tyrosine and tryptophan biosynthesi...
beta-Alanine metabolism
ABC transporters
Peroxisome
Purine metabolism
Valine, leucine and isoleucine degradation
Ribosome biogenesis in eukaryotes
RNA transport
Pyruvate metabolism
Other glycan degradation
0.02
0.03
0.04
0.05
0.06
Rich Ratio
Gene Number
3
7
12
16
20
Qvalue
0.00
0.02
0.04
0.06
b
Glycolysis / Gluconeogenesis
Pentose phosphate pathway
Galactose metabolism
Flavonoid biosynthesis
Folate biosynthesis
Arachidonic acid metabolism
Linoleic acid metabolism
Fructose and mannose metabolism
Photosynthesis
Purine metabolism
Starch and sucrose metabolism
Indole alkaloid biosynthesis
Phenylpropanoid biosynthesis
Stilbenoid, diarylheptanoid and gingerol biosynthe...
Carbon fixation in photosynthetic organisms
Glycerolipid metabolism
Plant hormone signal transduction
RNA polymerase
Pyrimidine metabolism
Carbon metabolism
0.005
0.010
0.015
0.020
0.025
Rich Ratio
Gene Number
1
2
2
3
3
Qvalue
0.1
0.2
0.3
0.4
0.5
c
Phosphonate and phosphinate metabolism
Glycerophospholipid metabolism
Protein processing in endoplasmic reticulum
Cysteine and methionine metabolism
Glycerolipid metabolism
Terpenoid backbone biosynthesis
Nucleotide excision repair
Endocytosis
Ribosome biogenesis in eukaryotes
Purine metabolism
Pyrimidine metabolism
RNA polymerase
Spliceosome
Pentose and glucuronate interconversions
RNA transport
MAPK signaling pathway - plant
0.02
0.04
0.06
Rich Ratio
Gene Number
1
2
2
3
3
Qvalue
0.2
0.4
0.6

Fig. S2 Scatter plot of the KEGG pathway enrichment of the three modules from WGCNA. a: KEGG pathways based on the MEred module. b: KEGG pathways based on the MEgreenyellow module. c: KEGG pathways based on the MEpurple module.
